# Ancient Humans Influenced the Current Spatial Genetic Structure of Common Walnut Populations in Asia

**DOI:** 10.1371/journal.pone.0135980

**Published:** 2015-09-02

**Authors:** Paola Pollegioni, Keith E. Woeste, Francesca Chiocchini, Stefano Del Lungo, Irene Olimpieri, Virginia Tortolano, Jo Clark, Gabriel E. Hemery, Sergio Mapelli, Maria Emilia Malvolti

**Affiliations:** 1 Institute of Agro-environmental and Forest Biology, National Research Council, Porano, Terni, Italy; 2 U.S.D.A. Forest Service, Hardwood Tree Improvement and Regeneration Center, Department of Forestry and Natural Resources, Purdue University, West Lafayette, Indiana, United States of America; 3 The Institute of Archaeological and Monumental Heritage, National Research Council, Tito Scalo, Potenza, Italy; 4 Earth Trust, Little Wittenham, Abingdon, Oxfordshire, United Kingdom; 5 Sylva Foundation, Little Wittenham, Oxfordshire, United Kingdom; 6 Institute of Agricultural Biology and Biotechnology, National Research Council, Milan, Italy; Estonian Biocentre, ESTONIA

## Abstract

Common walnut (*Juglans regia* L) is an economically important species cultivated worldwide for its wood and nuts. It is generally accepted that *J*. *regia* survived and grew spontaneously in almost completely isolated stands in its Asian native range after the Last Glacial Maximum. Despite its natural geographic isolation, *J*. *regia* evolved over many centuries under the influence of human management and exploitation. We evaluated the hypothesis that the current distribution of natural genetic resources of common walnut in Asia is, at least in part, the product of ancient anthropogenic dispersal, human cultural interactions, and afforestation. Genetic analysis combined with ethno-linguistic and historical data indicated that ancient trade routes such as the Persian Royal Road and Silk Road enabled long-distance dispersal of *J*. *regia* from Iran and Trans-Caucasus to Central Asia, and from Western to Eastern China. Ancient commerce also disrupted the local spatial genetic structure of autochthonous walnut populations between Tashkent and Samarkand (Central-Eastern Uzbekistan), where the northern and central routes of the Northern Silk Road converged. A significant association between ancient language phyla and the genetic structure of walnut populations is reported even after adjustment for geographic distances that could have affected both walnut gene flow and human commerce over the centuries. Beyond the economic importance of common walnut, our study delineates an alternative approach for understanding how the genetic resources of long-lived perennial tree species may be affected by the interaction of geography and human history.

## Introduction

Common walnut (*Juglans regia* L.) is a wind-pollinated, monoecious, long-lived, perennial tree cultivated throughout temperate regions worldwide for its timber and edible nuts [1]. In its Asian native range (from Xinjiang province of Western China to the Caucasus through Central Asia) *J*. *regia* survives and grows spontaneously in almost completely isolated stands surrounded by arid continental lowland, mountain slopes and highland steppes [2]. Evidence from the fossil pollen record indicates that *J*. *regia* occupied these niches since the Pleistocene glaciations [3]. Subsequently, barriers to gene flow, such as the Hindu Kush, Pamir, Tien Shan and Himalaya Mountains, and the progressive desertification of Central Asia during the Holocene promoted the fragmentation and isolation of natural *J*. *regia* populations in Asia [3].

Despite this natural geographic isolation, *J*. *regia* and other long-lived perennial tree fruit species evolved under the influence of human management and exploitation [4]. Consequences of human manipulation vary across species because, in general, plant cultivation and domestication is a spatially and temporary dynamic multi-stage process that results in populations ranging from exploited wild plants to cultivated forms that cannot survive without human intervention [5–7]. Typically, the domestication of perennial species has resulted in fundamental changes in the mode of reproduction (clonal propagation) and inflorescence / fruit characteristics [4, 8]. *Juglans regia*, however, does not meet this broadly endorsed criteria for domestication [9, 10], as its cultivated forms are not essentially different from wild, autochthonous trees. Cultivated walnuts are likely derived from selection of seedlings from geographically distinct natural populations over the course of many thousands of years [10, 11].

Indeed, *J*. *regia* has been closely associated with human activities since the Early Bronze Age in Asia. Fossilized remnants of desiccated walnut seeds have been found in three macro-regions recognized as primary centers of early fruit tree cultivation [12]: the Near-East (Southern Armenia, Areni-1 Cave, 6230–5790 yr. BP) [13], Central Asia (Kashmir Valley, Pakistan, Kanispur, 5149 yr. BP) [14] and northeastern China along the Yellow River basin (Hebei Province, Chishan, 7300 yr. BP) [15]. Vahdati [16] described *J*. *regia* as an ancient tree food whose use has been tightly related to the religious beliefs, history and local identity of rural communities. It was constantly traded via networks such as the Persian Royal Road [17] and the Silk Roads [18] that connected China and India to Mediterranean regions. These roads linked culturally dissimilar pastoralist and agrarian civilizations from different parts of Eurasia and allowed the exchange of technologies, goods, religions, languages, ideas and agricultural products, resulting in a rich economic and technological synergy that promoted the rise of modernity over several millennia [19].

The emerging field of plant bio-cultural diversity integrates cultural features that identify distinct human ethnic groups, such as language, life habits, and food, with plant diffusion and traditional seed-management practices / exchanges [20]. For example, there is a close relationship between ethnolinguistic diversity—used as a proxy for human cultural interactions—and the spatial genetic structure of some maize (*Zea mays*) [21] and sorghum populations (*Sorghum bicolor* L.) [22]. A preliminary genetic analysis of *J*. *regia* from Yunnan province (China) revealed that village networks and familial relationships contributed to the genetic structure of autochthonous populations of walnut [23]. In light of these findings, we expected that longstanding human contact with walnut, an economically and culturally significant food source that was widespread, highly nutritious, easily harvested, transported and consumed (requiring no special knowledge to grow or cook),–will have affected the spatial genetic structure of *J*. *regia* in Asia.

In the present study we evaluate the hypothesis that the current distribution of autochthonous populations of common walnut in Asia is the product of ancient anthropogenic dispersal and human cultural interactions. In particular, we draw on linguistic and anthropological evidence to determine if (i) major ancient trade routes such as Silk Roads acted as “gene corridors”, facilitating human-mediated gene flow among autochthonous common walnut populations in Asia, and (ii) the presence of ethno-linguistic barriers, reflecting cultural differences among human communities, influenced the genetic structure of autochthonous *J*. *regia* populations in Asia.

## Results

### Silk Roads and the spatial genetic structure of common walnut populations in Asia

**Fig 1:**
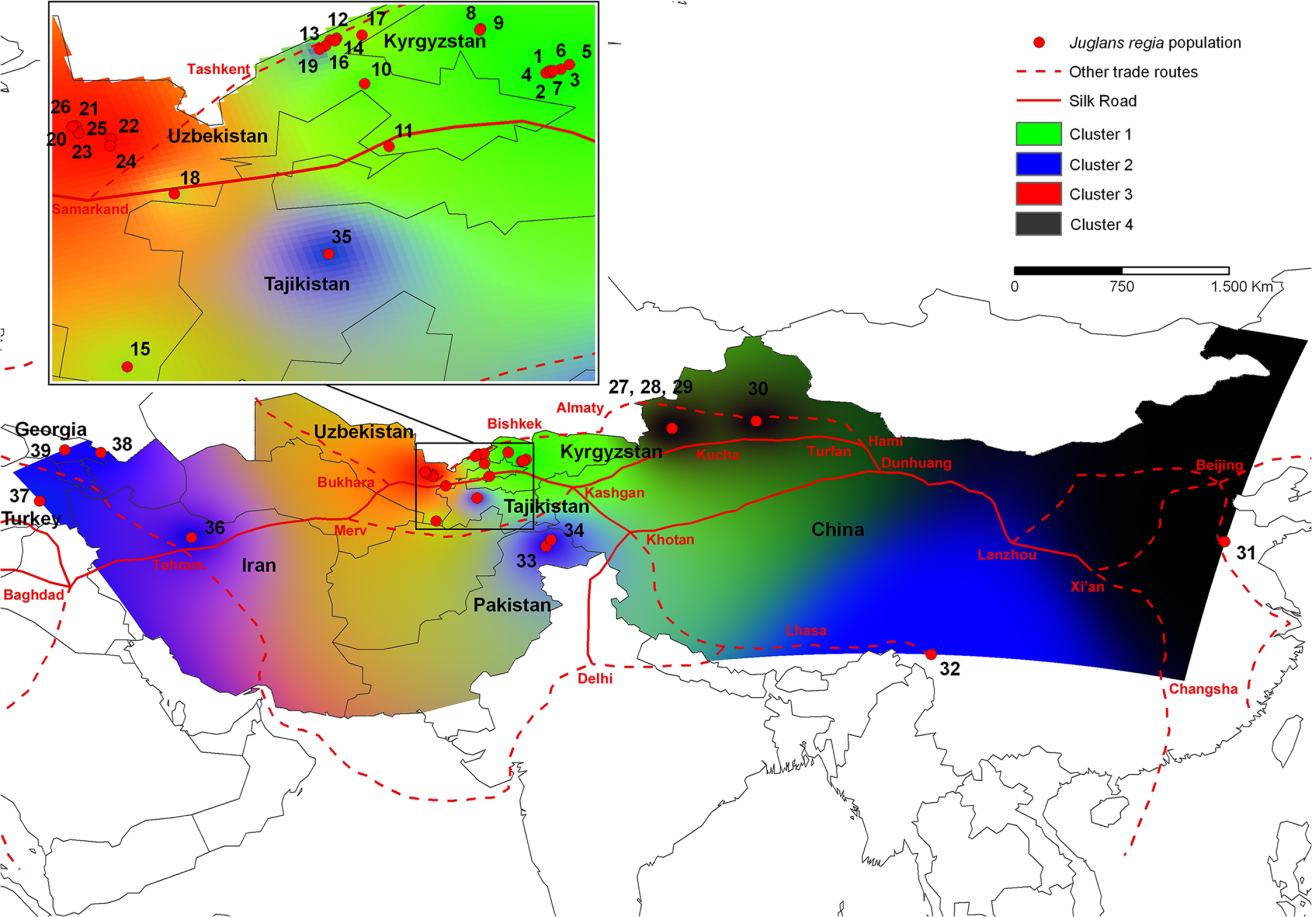
Spatial genetic structure of walnut populations and trade routes across Asia. Synthetic map of IDW interpolations of the estimated mean population membership values (*Qi*) in the K = 4 clusters inferred by STRUCTURE [24] for 39 common walnut populations (red dots) in the species’ Asian range. The Silk Road (solid red line) and other trade routes (dotted red line) across the Asian continent were reported as proposed by Francis et al. [25]. Details concerning common walnut populations are listed in S1 Table.

As reported in Pollegioni et al. [3], STRUCTURE clustering analysis [24] recognized K = 4 as the best representation of the underlying hierarchical structure of the 39 common walnut populations in Asia. In this current study, the synthetic map generated by superimposing the four genetic cluster’s *Q*—surface maps on the map of the Silk Road [25] showed that cluster 1 comprised all nine Kyrgyz populations (1-Ak-Terek, 2-Sharap, 3-Yaradar, 4-Shaidan, 5-Kyzyl-Ungur, 6-Katar-Yangak, 7-Kyok-Sarau, 8-Kyr, 9-Ters-Kolt) sampled in the walnut forests of the Western Tien Shan mountains located near the Fergana Valley (Q_1_ ≥ 0.8956) (Fig 1, S1 and S2 Tables). Cluster 2 centered in Western and South-Central Asia, included all walnut samples from three Trans-Caucasus sites (37-Anatolia, Turkey; 38-Lagodekhi, 39-Skra, Georgia), Alborz ridges, Iran (36-Karaj), northern Pamir ridges, Tajikistan (35-Shouli), Kashmir-western Himalayas, Pakistan (33-Gilgit Valley and 34-Hunza Valley) and the Tibetan-eastern Himalaya, China (32-Dashuicum) (Q_2_ ≥ 0.8001) (Fig 1, S1 and S2 Tables). In addition, 21 walnut trees (58.3%) collected in 19-Karankul (eastern Uzbekistan) and ten of 67 walnut trees (14.9%) from 28-Gongliu-2 (Xinjiang province, China) were unambiguously assigned to cluster 2 (Q_2_ ≥ 0.800). Cluster 3 assembled all seven populations from Nurata ridges (20-Farish, 21-Andigen, 22-Katta-Bogdan, 23-Khayat, 24-Yamchi, 25-Karri, 26-Madjerum) located in east-central Uzbekistan ~150 km north-west of Samarkand (Q_3_ ≥ 0.9363). Cluster 4 included sites from northern and eastern China, i.e., four sites in the Eastern Tien Shan mountains, Xinjiang province, (Gongliu Wild Walnut Nature Reserve 27-Gongliu-1, 28-Gongliu-2, 29-Gongliu-3 and Urumqi County 30-Urumqi) and one site from Shandong province, (31-Sunbè) (Q_4_ ≥ 0.8001) (Fig 1, S1 and S2 Tables). The northern route of the Northern Silk Road originated from the historical capital of Chang’an (now Xi’an, Xhaanxi province), ran through Gansu Province via Lanzhou and Dunhuang along the Hexi Corridor. This road went westward along the northern foot of the Eastern Tien Shan mountains. It connected Shandong province and Urumqi, allowing the exchange of shelled (e.g. walnut, pistachio) and stone (e.g. apricot, peach) fruits among pastoralist and agrarian civilizations [19] (Fig 1, S1 Fig). The remaining six *J*. *regia* populations in the Western Tien Shan mountains (10-Kamchik, 12-Sidjak, 13-Charvak, 14-Nanai, 16-Bogustan and, 17-Bostanlyk) and two populations from the Fergana Valley (11-Yakkatut), and Gissar mountains (15-Djarkurgan) in eastern Uzbekistan were mainly admixtures among cluster 1 (0.4842 ≤ Q_1_ ≥ 0.7780) and cluster 3 (0.1180 ≤ Q_3_ ≥ 0.3250) with *Q*-predominance of cluster 1. The 18-Bakhmal population sampled in the Zaamin mountains was also admixed combining genetic elements of clusters 1, 2 and 3 (Q_1_ = 0.4401, Q_2_ = 0.1507, Q_3_ = 0.3525). These admixed *J*. *regia* populations are from East-Central Uzbekistan where the northern and central routes of Northern Silk Road converged (Fig 1, S1 Fig, S1 and S2 Tables).

**Fig 2:**
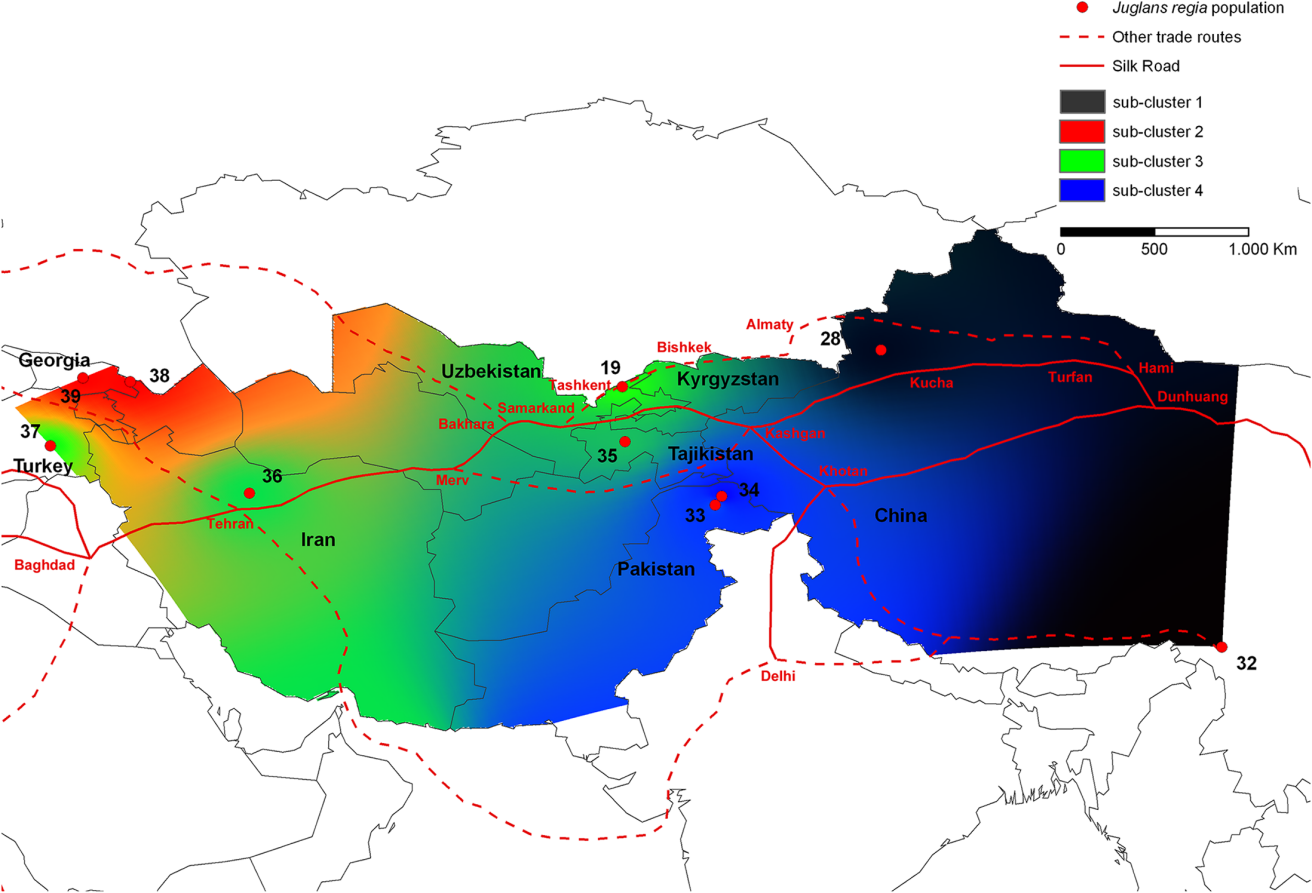
Spatial genetic sub-structure of walnut cluster 2 and trade routes across Asia. Synthetic map of IDW interpolations of the estimated mean population membership values (*Qi*) in the K = 4’ sub-clusters of cluster 2 inferred by STRUCTURE [24] (19-Karankul, 28-Gongliu-2, 32-Dash, 33-Gilgit, 34-Hunza, 35-Shouli, 36-Karaj, 37-Anatolia, 38-Lagodekhi, 39-Skra). The Silk Road (solid red line) and other trade routes (dotted red line) across the Asian continent were reported as proposed by Francis et al. [25].

Subsequent STRUCTURE analysis within each of the previously inferred clusters did not reveal any genetic substructure except for cluster 2 which was divided into four sub-clusters (K’ = 4). These four sub-clusters divided walnut trees of 28-Gongliu-2 (ten samples, Xinjiang province, China,) and 32-Dashuicum (Tibet, China) (sub-cluster 1) from 38-Lagodekhi, 39-Skra (Georgia) (sub-cluster 2), 37-Anatolia (Turkey), 36-Karaj (Iran), 35-Shouli (Tajikistan), 19-Karankul (21 samples, Eastern Uzbekistan), (sub-cluster 3) and 33-Gilgit Valley and 34-Hunza Valley (Kashmir, Pakistan) (sub-cluster 4) (Fig 2, S2 Table). The four geographically distant populations of sub-cluster 3 were joined by the westernmost section of the Silk Road, specifically the Northern Silk Road heading west from Samarkand and Bukhara (where the northern, central and southern routes joined), to the Mediterranean Sea (Fig 2, S1 Fig). The UPGMA tree based on Nei’s [26] genetic distances confirmed the previous results and divided 39 *J*. *regia* populations in four main clusters and four sub-clusters (S2 Fig).

### Correlation between population structure of common walnut and human linguistic diversity

We observed a positive trend between genetic distances (D_GEN_) among *J*. *regia* populations and linguistic distance (D_LAN_) among human communities living in the 39 Asian sampling sites. One-way analysis of variance (ANOVA) and the subsequent post hoc Tukey’s tests revealed statistically significant differences in the mean pairwise *F*_ST_ [27] and *D*_est_ [28] among four linguistic classes (*P* < 0.0001). An increase of mean genetic distance of common walnut was associated with an increase of mean linguistic distance among human communities, varying from *F*_ST_ = 0.085 ± 0.056 and *D*_est_ = 0.081 ± 0.068 for the category D_LAN_ = 0 (same language) to *F*_ST_ = 0.1848 ± 0.049 and *D*_est_ = 0.280 ± 0.076 for the category D_LAN_ = 4 (different phyla) (S3 Fig).

Both geographic (D_GEO_) and human linguistic variables (D_LAN_) appeared significantly correlated with walnut genetic diversity (D_GEN_) calculated using either *F*_ST_ or *D*_est_ statistics (Table 1A). In particular, the pairwise linearized genetic differentiation values [*F*_ST_ /(1-*F*_ST_)] or [*D*_*est*_ /(1-*D*_est_)] and the natural logarithm of geographic distances (straight-line distances in km) among sampling sites were significantly correlated (*r* (*F*_ST_) = 0.6248, *P* = 0.0002; *r* (*D*_est_) = 0.6521, *P* = 0.0002). Space (latitude, longitude) and human interactions may act simultaneously on gene flow, however, influencing the genetic structure of common walnut populations. Simple Mantel tests [29, 30] revealed that human linguistic diversity was positively correlated with pairwise genetic divergence of *J*. *regia* populations (linearized *F*_ST_; *r* = 0.4974, *P* = 0.0002; linearized *D*_est_; *r* = 0.6493 *P* = 0.0002) and straight-line geographic distances (*r* = 0.5397, *P* = 0.0002) among common walnut populations (Table 1A). Thus, the observed relationship between D_GEN_ and D_LAN_ matrices might have occurred as a result of a common spatial component. After controlling for the effect of geographic distances (natural logarithm of straight-line) among populations, the partial correlation of linearized *F*_ST_ and D_LAN_ matrices remained significant but low (partial Mantel test *r* = 0.2012, *P* = 0.0084). However, when *D*_est_ was used as a measure of genetic distance among walnut populations, the partial correlation between human linguistic distances and *J*. *regia* genetic diversity remained significant and high even after the effect of D_GEO_ matrix was held constant (partial Mantel test *r* = 0.4297, *P* = 0.0002) (Table 2A). The Multiple Regression on distance Matrices (MRM) analysis [31] indicated that the effects of geographic distances (D_GEO_) and human linguistic diversity (D_LAN_) on SSR genetic divergence among 39 common walnut populations in Asia (D_GEN_) were significantly positive using *F*_ST_ (standardized partial regression coefficient, ß_GEO_ = 0.0303, *P* = 0.0002; ß_LAN_ = 0.0118, *P* = 0.0116) and *D*_est_ (ß_GEO_ = 0.0295, *P* = 0.0002; ß_LAN_ = 0.0296, *P* = 0.0116) (Table 1B). The MRM model showed that geographic and language distance together explained 41.50% (*P* = 0.0002) and 53.13% (*P* = 0.0002) of the *F*_ST_- and *D*_est_-estimates, respectively (Table 1B).

**Table 1:** Correlation between genetic distances among walnut populations and human linguistic distances.

	Genetic differentiation coefficient ^a^			
	F_ST_		D_*est*_	
(A) Mantel test ^b^	Correlation coefficient *(r)* ^d^	Proportion of variance explained *(r* ^*2*^ *)*	Correlation coefficient *(r)* ^d^	Proportion of variance explained *(r* ^*2*^ *)*
D_GEN_ x D_GEO_	0.6248***	0.3904	0.6521***	0.4252
D_GEN_ x D_LAN_	0.4974***	0.2474	0.6493***	0.4215
D_GEO_ x D_LAN_	0.5397***	0.3525	0.5397***	0.3525
(D_GEN_ x D_LAN_) •D_GEO_ ^c^	0.2012**	0.0405	0.4297***	0.1846
	F_ST_		D_*est*_	
(B) MRM ^b^	Coefficient of Regression (ß) ^e^	*r* ^2^	Coefficient of Regression (ß) ^e^	*r* ^2^
Intercept	-0.0294	0.4150***	-0.0562	0.5313***
D_GEO_	0.0303***		0.0295***	
D_LAN_	0.0118*		0.0296***	

^a^ Measures of genetic differentiation calculated among 39 common walnut populations using either F_ST_ [27] and D_*est*_ [28].

^b^ (A) Simple and Partial Mantel tests [29, 30] and (B) Multiple Regression Model analysis [31] of genetic (D_GEN_) on geographic (D_GEO_) and linguistic (D_LAN_) matrices.

^c^ Partial correlation coefficient.

^d^ Significance of *r* values was tested using 5000 permutations as implemented in ZT software [59]: * P < 0.05, ** P < 0.01 and *** P < 0.001.

^e^ *P* values are based on 5000 permutations as implemented in R Ecodist package [61]: * P < 0.05, ** P < 0.01 and *** P < 0.001.

**Table 2:** Delaunay connections associated with linguistic distance (D_LAN_) and crossed by a statistically significant genetic barrier.

	Delaunay connections		
Linguistic distance	Crossed by a genetic barrier ^a^	Not crossed by a genetic barrier	Total
D_LAN =_ 0	10 (15.4%)	55 (84.6%)	65
D_LAN =_ 1	0	0	0
D_LAN =_ 2	3 (37.5%)	5 (62.5%)	8
D_LAN =_ 3	4 (66.7%)	2 (33.3%)	6
D_LAN =_ 4	24 (100%)	0 (0%)	24
Total	41(39.80%)	62 (60.2%)	103

^a^ Statistically significant genetic barriers were calculated using the Monmonier’s maximum difference algorithm as implemented in BARRIER software 2.2 [62].

Assuming that human linguistic similarities affected the spatial dispersal of *J*. *regia* resources in Asia, we expected the pairwise linguistic differences between human communities on opposite sides of a walnut genetic barrier to be higher than the linguistic differences on the same side of the barrier. In a previous analysis of this dataset [3], five statistically significant genetic barriers among 39 common walnut populations were identified (S4 Fig). In this study, we found that out of the 103 Delaunay connections associated with linguistic distance, 41 (39.80%) crossed significant genetic barriers. All 24 of the Delaunay connections (100%) between linguistic phyla (D_LAN_ = 4) were crossed by significant genetic barriers, but only 17 connections (21.15%) within linguistic phyla (D_LAN_ = 0, 1, 2, 3) were crossed by genetic barriers (Table 2). There was a significance difference (*X* ^*2*^ = 44.05, *P* < 0.001) between the percentage of Delaunay connections “between” and “within” linguistic phyla that were crossed by statistically significant genetic barriers. Thus, stronger genetic barriers of common walnut were significantly associated with larger linguistic differences between sampled sites.

**Fig 3:**
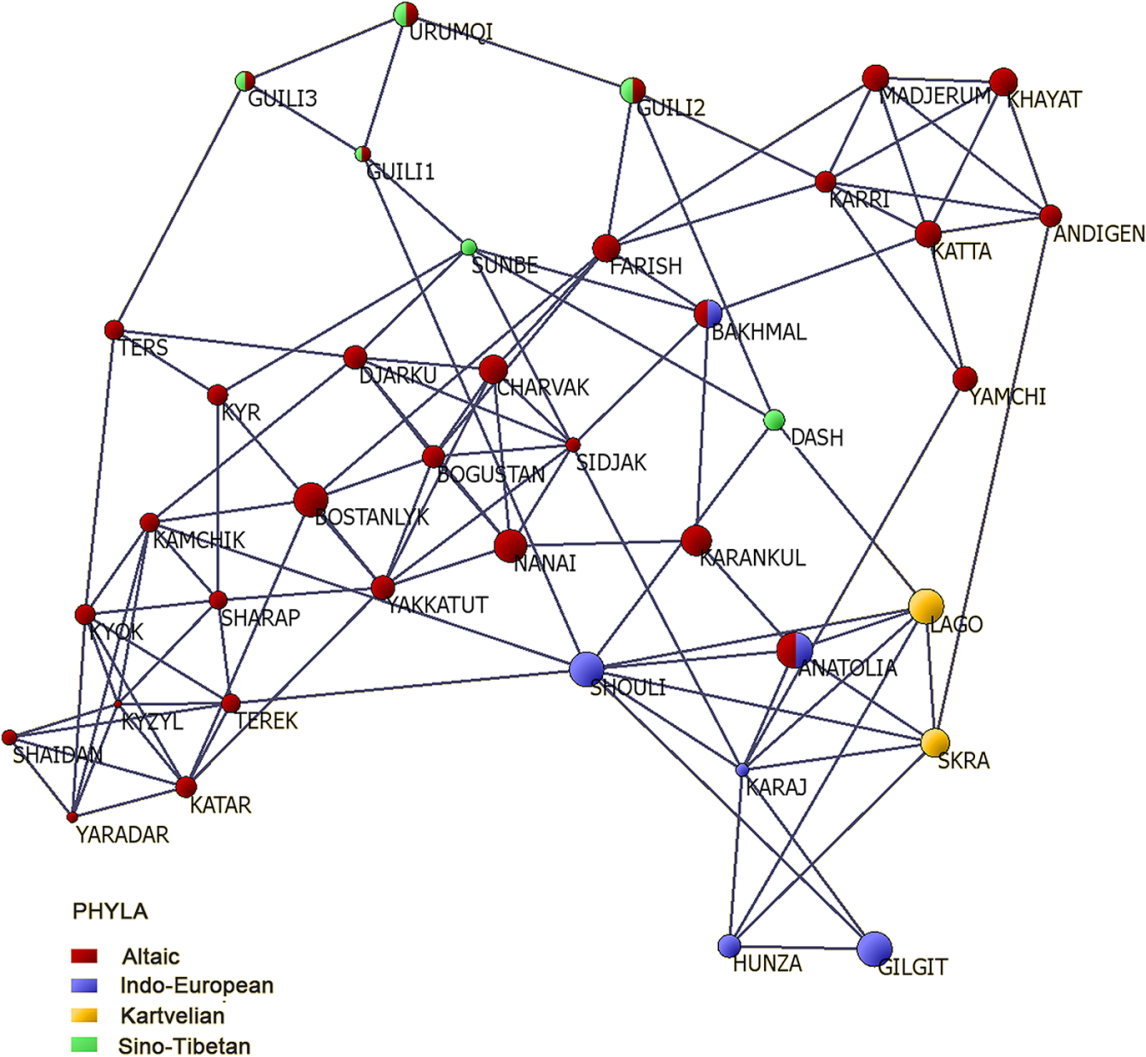
Common walnut population graph for 39 study sites in the Asian range. Nodes represent geographic sites with diameter proportional to within-site heterozygosity and length of edges connecting nodes equivalent to genetic differentiation among the sites calculated using 14 SSR markers. The color of each node represents the language phylum spoken by human communities living in the geographic sampling sites.

A multivariate population graph displayed a partial spatial coincidence between the inferred population structure of *J*. *regia* and the linguistic diversity detected among human communities living in the sampled sites (Fig 3). In particular, five (33-Gilgit Valley, 34-Hunza Valley, Kashmir, Pakistan; 35-Shouli, Tajikistan;36-Karaj, Iran; 37-Anatolia, Turkey) of the nine *J*. *regia* populations included in cluster 2 were located in sites where Indo-European speakers are predominant. Three distinct linguistic phyla, Sino-Tibetan, Kartvelian and Altaic, are prevalent in the remaining four *J*. *regia* sites of cluster 2, 32-Dashuicum (Tibetan and Chinese-Mandarin, China), 38-Lagodekhi, 39-Skra (Georgian, Georgia) and 19-Karankul (Northern Uzbek, Eastern Uzbekistan), respectively (Fig 3). Current speakers of Turkic languages (Altaic phylum) were mainly localized in Western Kyrgyzstan and East-Central Uzbekistan, corresponding to the geographic distribution of genetic cluster 1 (Kyrgyz language; 1-Ak-Terek, 2-Sharap, 3-Yaradar, 4-Shaidan, 5-Kyzyl-Ungur, 6-Katar-Yangak, 7-Kyok-Sarau, 8-Kyr, 9-Ters-Kolt), cluster 3 (Northern Uzbek language; 20-Farish, 21-Andigen, 22-Katta-Bogdan, 23-Khayat, 24-Yamchi, 25-Karri, 26-Madjerum), and their admixed populations (Northern Uzbek language; 10-Kamchik, 11-Yakkatut, 12-Sidjak, 13-Charvak, 14-Nanai, 15-Djarkurgan, 16-Bogustan, 17-Bostanly) (Fig 3). The population sampled in Bakhmal showed a complex pattern of genetic admixture that included clusters 1, 2 and 3. Bakhmal is located in the Jizakh province of Central Uzbekistan where Northern Uzbek (Turkic) and Tajiki (Indo-Iranian) are currently spoken. A co-distribution of the Sino-Tibetan language phylum and walnut genetic cluster 4 (27-Gongliu-1, 28-Gongliu-2, 29-Gongliu-3, 30-Urumqi, 30-Sunbe’) of Western and Eastern China was also detected (Fig 3).

## Discussion

Any phylogeography of common walnut and any reconstruction of its Holocene distribution must account for its long history of human use and the clear historical record of human dispersal of walnut over the past millennium [16–18, 32, 33]. The evidence that dispersal by ancient humans shaped the current spatial genetic structure of autochthonous populations of common walnut in Asia comes from the integration of genetic data with historical and linguistic sources. For walnut, as with any food plant, these factors are necessarily interdependent. Two measures of common walnut genetic differentiation (*D*_*est*_ and *F*_*st*_) were both positively and significantly correlated with divergence in human language phylogeny, even after accounting for the effects of geographic distance between sampled sites (Table 1). Furthermore, analysis of barriers showed that spatial genetic partitions (typically interpreted as barriers to gene flow) coincided with large differences in human language (Table 2). Conversely, similarities in human language over large geographic areas facilitated the dispersal of walnut, its introduction to new habitats, and the genetic homogenization of disparate populations.

As an example of the interrelationship between language, cultural history, geography, and the distribution of common walnut, consider genetic cluster 4 (Fig 1), which groups four Chinese sites in the Eastern Tien Shan mountains and a population from Shandong, eastern China. This walnut genetic cluster overlaps substantially the distribution of the Chinese-Mandarin language (Sino-Tibetan phylum). The earliest designation for common walnut in ancient Chinese-Mandarin is *Hú táo* 胡桃 (peach of the Hú people) [34]. According to Ashuri [35], *Hú* represented the Xiongnu nomads that formed a great tribal league at the end of the 3rd century BCE. They became a dominant force across Southern Siberia Mongolia, Gansu and Xinjiang by defeating the Indo-European Yuezhi tribes who then migrated from the Tian-Shan range and Tarim basin to Sogdiana in 176 BCE [36]. The Natural Science Annals of Zhang Hua (232–300 CE) reported that the Chinese diplomatic emissary Zhang Qian was sent to Central Asia by the Emperor Wudi in 138 BCE and 119 BCE via the ancient track connecting the imperial capital Xi’an to Urumqi. His missions, to negotiate a military alliance with the Yuezhi against the Xiognu, represented the first steps in the development of the northern route of the Silk Road [19]. *Hú táo* likely alludes to the source of *J*. *regia* (Xiongnu tribes in Xinjiang during the Han dynasty) introduced into East China from Central Asia along the Silk Road, as has been corroborated by our genetic analyses.

Commonality of language likely contributed to the mixing of *J*. *regia* gene pools as well as their dispersal to new habitats. In Western Kyrgyzstan and East-Central Uzbekistan *J*. *regia* grows where two Turkic languages of the Altaic phylum are spoken, i.e., Kyrgyz and Northern Uzbek. The Old Turkic word for walnut was *jaɣaq* (Orkh.), a word borrowed from the Proto-Turkic etymologic root *jAŋgak [37]. This old Turkic word can be recognized in many modern Turkic languages including Kyrgyz (ǯaŋaq or *ǯaŋɣaq*) and Northern Uzbek (*jɔŋɣɔq*) [37]. Therefore, despite the presence of substantial physical barriers to gene flow (e.g., the Kyzyl Kim desert, the Tien Shan mountains), both the dissemination of the Old Turkic word *jaɣaq* for walnut and the admixed SSR profiles of common walnut trees in Eastern Uzbekistan and populations from the Fergana Valley and Gissar mountains indicate the exchange of *J*. *regia* among Turkic communities that lived between Tashkent and Samarkand where the northern and central routes of the Northern Silk Road converged (Fig 1).

The spatial pattern of five widely separated common walnut populations of genetic cluster 2 sampled from Western and Central Asia (Tajikistan, Iran, Turkey, Pakistan) coincided with the geographic distribution of the Iranian (Tajiki, Persian-Iranian), Anatolian, and Indo-Aryan (Urdu) language families of the Indo-European phylum. In the Persian-Iranian language and its chief dialect (Tajik), common walnut is called *gerdoo* or *gôz* (an archaic form of *gerdoo*). According to Hasandust [38], the etymologic root of *gôz* corresponds to the Old Persian word *angawza> *angawz> *gawz* (~ 500 BCE), meaning something hidden inside a shell. Laufer [34] also recognized *gawz* as the Iranian base for walnut as reflected in the word *koz* in Turkish-Anatolian, *akhrot* in Urdu, אגוזא in Aramaic and *ywš* in Sogdian. Aramaic, Sogdian, and subsequently Middle Persian became the “trade” languages and lingua franca of the Persian Royal Road and the Silk Road connecting Western Asia to Central Asia [39]. Therefore, the genetic similarity of walnut populations sampled in Western and Central Asia and the wide dissemination of the Persian root *gawz* lead us to conclude that walnut management and the Persian language co-dispersed through long-distance trade across the Persian Empire starting from the Achaemenid phase (500–330 BCE) (co-dispersal hypothesis). Fossil pollen evidence also indicates that the establishment of the Middle Persian and Achaemenid Empires corresponds with the maximum expansion of walnut cultivation across Irano-Turanian regions [40].

Cultural barriers represented by linguistic dissimilarity, in conjunction with evolutionary processes such as selection and drift, were nevertheless, in some cases, sufficiently strong to constrain the genetic homogenization of walnut by ancient commerce in Asia. For example, 35-Shouli is a population in sub-cluster 3, sampled from the Pamir range in Tajikistan (Fig 2). It is more closely related genetically to 37-Anatolia (southwestern Turkey), a population ~3,000 km away (but near the westernmost section of the Silk Road that leads from Baghdad to the Mediterranean) than to 11-Yakkatut, an Uzbek population adjacent to the northern branch of the Silk Road and only a few hundred kilometers from Shouli. This barrier of language appears to have isolated walnut trees from Shouli from those in Yakkatut, even though these populations are geographically in close proximity, and linked them to populations from Anatolia, far distant.

Our findings demonstrate that although common walnut is considered a Tertiary relict in Central Asia [41], the extent to which isolated, apparently autochthonous populations of *J*. *regia* in Asia are anthropogenic is unresolved. It is likely that some, and possibly many, populations were planted thousands of years ago, which helps explain the link between genetic diversity and language phylogeny that we observed. Records of tree planting in Asia, which often followed the preparation of land with fire, date to at least 1100 BCE [42]. Historical sources attest that common walnut was used extensively for nut production and re-forestation / afforestation in Western (e.g. Georgia, Iran and Turkey) and Central Asia (e.g. Uzbekistan, Tajikistan, Kyrgyzstan) since at least the 5th century CE [43, 44].

Pome fruits, olives, almonds, pistachios, pomegranates, and common walnut are among the many perennial food plants dispersed across Asia by human migration, merchants, armies and imperial emissaries [33]. Of these species, common walnut is unusual because only recently (within the past century) has been widely propagated by grafting, and it is still propagated almost exclusively by seed across Asia [45]. Why did selection and domestication (including propagation by grafting), which affected other perennial crops’ morphology and distribution so dramatically, not obliterate the genetic signal of dispersal in common walnut? It is possible that the genetic variability in *J*. *regia* was already low by the time humans began to use it or that walnuts used for trade and afforestation by ancient cultures had already experienced a genetic bottleneck, perhaps due to selection for large, light-colored kernels and thin shells [41]. However, all populations we sampled which spanned thousands of kilometers are nearly indistinguishable morphologically, especially with regard to shell thickness and percent kernel (Malvolti M.E, Mapelli S. personal observations). An important feature of common walnut that may also have contributed to its dispersal, planting and maintenance is the high quality of its wood. Walnut grows to a large mature size and produces not only edible fruits, but also wood that has always been prized for its strength, luster and workability. The value of its wood likely contributed to common walnut’s dispersal by humans and its use in afforestation. However, remains of *J*. *regia* wood in archaeobotanical record are rare. Little robust evidence exists to support the idea that differences in planting or harvesting strategies for timber versus fruit production occurred across Central-Western Asia. Nevertheless, there are some data related to the use of common walnut wood by ancient cultures. Artifacts and remnants made of walnut wood were found in graves at Uruk dated to Neo-Babylonian period (Iraq, ~626–529 BCE) [45] and from the royal tomb at Gordion, capital of the Phrygian state and a major trade center in Central Anatolia between 950 and 550 BCE [46]. The second-oldest known diptych has found at the ancient Assyrian city Nimrud (Iraq, 8^th^ century BCE) and was constructed of walnut [47]. Thus, common walnut was unusual among Asian perennial crops because the value of its wood and the value of walnut wood probably contributed to its spread beyond orchards, making it a permanent feature of Asian landscapes.

## Materials and Methods

### Genetic dataset

To represent the genetic diversity of *J*. *regia* in Asia, we analyzed a published dataset comprising 39 autochthonous common walnut populations (926 total genotypes) sampled from China, Kyrgyzstan, Uzbekistan, Tajikistan, Pakistan, Iran, Turkey and Georgia growing in eight mountain ranges (Tien Shan, Gissar, Zaamin, Nurata, Pamir, Himalayas, Alborz, Trans-Caucasus). These were genotyped using 14 unlinked nuclear, neutral microsatellite (SSR) markers [3] (S1 Fig, S1 Table). Potential sources of bias from the selection of the 14 SSR markers (e.g. selective pressure, presence of null alleles) affecting the genetic structure of walnut populations were evaluated and ruled out by Pollegioni et al [3].

### Language classification of human communities

The human communities that live at the 39 sampled sites and speak ten languages (Chinese-Mandarin, Uyghur, Tibetan, Kyrgyz, Northern Uzbek, Tajiki, Urdu, Persian-Iranian, Turkish, and Georgian) which were classified into four linguistic phyla (Altaic, Indo-European, Sino-Tibetan and, Kartvelian), seven linguistic families (Turkic, Iranian, Sinitic, Tibeto-Burman, Indo-Aryan, Anatolian and, Karto-Zan) and six linguistic subgroups (Western-Turkic, Eastern-Turkic, Southern-Turkic, Western Iranian, and Central Indic) based on two sources, The Ethnologue website [48] and Ruhlen’s [49] (S1 Table). Although a universally accepted taxonomy of human languages is not recognized, Ruhlen’s classification has been extensively applied in genetic studies of human populations [50, 51]. Allowing for differences in linguistic phylogeography, we assigned the language of each sampling site using two sources, with only one exception: the Indo-European phylum of The Ethnologue website was replaced by the Indo-Hittite phylum of Ruhlen’s classification (S1 Table). We encountered difficulties defining the linguistic affiliation of six human communities (Gongliu-1, Gongliu-2, Gongliu-3, and Urumqi from Xinjiang, Bakhmal from Uzbekistan and Anatolia from Turkey) as they are unofficially bilingual. Uyghur is a Turkic language currently written in the Arabic script with about 10 million speakers mainly living in the Xinjiang Uyghur Autonomous Province of North-Western China. The former multilingualism and cultural pluralism of this region have been progressively curtailed in favor of a monolingual policy that favors Chinese-Mandarin [52]. Bakhmal is located in the Jizakh province of Central Uzbekistan bordering Tajikistan to the south-east. Both Northern Uzbek (the official language) and Tajiki (the local language) are currently spoken there [48]. Finally, Turkic speakers of Anatolia are descendants of indigenous Indo-European farmers who adopted Turkic only in the early second millennium CE [53]. During the 11th century CE, Turkic nomads such as Seljuks and Ottomans occupied the grassland in the interior of Asia Minor, imposing their language (Turkic) and replacing Anatolian, an extinct branch of the Indo-European family by an elite dominance process. Therefore, Anatolia was classified as a site with Altaic and Indo-European speakers (S1 Table).

### Data analysis

#### Genetic structure analysis of common walnut populations

Three complementary statistical approaches were used to analyze the influence of anthropogenic dispersal on the spatial genetic structure of *J*. *regia* populations in Asia. First, a fully Bayesian clustering approach implemented in STRUCTURE software 2.3.3 [24] was conducted to detect the most likely number of populations as described by Pollegioni et al. [3]. The groups inferred by the first STRUCTURE analysis were then reprocessed separately to identify the possible substructure (sub-clusters). After determining the most probable number of clusters, an arbitrary threshold of Q ≥ 0.80 was used to assign populations and/or genotypes to one group. Populations or individuals with 0.2 < Q < 0.8 were classified as admixed. Following the procedure of Pollegioni et al [3], we derived K continuous clustering surfaces by interpolation of the population membership *Q*-values for the K clusters estimated from STRUCTURE using Inverse Distance Weighted (IDW) interpolation implemented in ArcGIS 9.3 (ESRI, Redlands, Calif. USA). A synthetic map representing the genetic structure of common walnut in Asia was obtained by overlaying the computed K clustering surface maps. We combined multiple K interpolated raster bands in a single multiband raster dataset by the Composite Bands function implemented in ArcGIS 9.3. As described by Bucci et al [54], the integrated use of the Composite Bands-tool and RGB color code allowed us to display the inferred genetic clusters of *J*. *regia* populations. To evaluate the role of caravans in transferring common walnut seeds throughout the Asian continent, we projected the Silk Roads and other trade routes on the synthetic map as proposed by Francis et al. [25] using ArcGIS 9.3. To confirm the genetic repartition of common walnut populations inferred by STRUCTURE, a UPGMA (Unweighted Pair Group Method with Arithmetic mean) tree analysis was also constructed based on Nei’s [26] genetic distance. Bootstrap support for this tree was determined by resampling loci 1000 times using POPTREE2 software [55].

Finally, to quantify and visualize the genetic relationships among *J*. *regia* populations and simultaneously display the linguistic patterns of human communities in the sampled sites, a multivariate graph approach [56] was applied using POPGRAPH software (http://dyerlab.bio.vcu.edu/software.html). In the resulting graph, *n* common walnut populations were represented by *n* nodes with node size and color equivalent to within-site heterozygosity and the language phylum spoken by human communities living in the sampling sites, respectively. The length of edges connecting nodes was proportional to the among-site genetic differentiation. Nodes were connected by the minimum number of edges necessary to maintain the overall genetic covariance structure among populations [56].

#### Correlation between population structure of common walnut and human linguistic diversity

Two measures of genetic differentiation among the 39 common walnut populations (*d*_GEN_ matrices), *F*_ST_ [27] and *D*_*est*_ [28], were estimated across 14 SSR loci using Arlequin version 3.11 software [57] and the web-based software SMOGD 1.2.5 [58] respectively. Because the dependence of *F*_ST_ values on within-population heterozygosity can lead to an underestimation of the true level of genetic differentiation using highly polymorphic microsatellite markers, the unbiased estimator of Jost’s (*D*_*est*_) was used as an alternative measure of genetic differentiation among walnut populations. Linguistic distances among human communities living in the sampled sites were calculated as simple dissimilarity indexes ranging from 0 to 4 according to the *d*_LAN_ matrix method described by Belle and Barbujani [50]. Human populations speaking languages belonging to different phyla were assigned *d*_LAN_ = 4, languages of different families *d*_LAN_ = 3, languages of different subgroup *d*_LAN_ = 2, different languages *d*_LAN_ = 1 and the same language *d*_LAN_ = 0. One-way analysis of variance (ANOVA) was used to detect a statistical difference in the walnut genetic differentiation among five human linguistic distance-classes. Pairwise comparisons among linguistic classes using *D*_est_ and *F*_ST_ values was performed based on a post hoc Tukey’s test using XLSTAT2010 software (http://www.xlstat.com).

We tested the effect of geographic distances (*d*_GEO_) and human linguistic diversity (*d*_LAN_) on gene flow among common walnut populations (*d*_GEN_) using non-parametric pairwise simple and partial Mantel tests [29, 30]. The p-value for the Z-score of the Mantel association parameter was inferred using 5,000 permutations as implemented in ZT software [59]. Assuming a non-linear distribution of sampling sites, we first tested for isolation by distance between populations (IBD) by regressing Slatkin’s linearized [*F*_*ST*_ / (1- *F*_*ST*_)] and [*D*_*est*_ / (1- *D*_*est*_)] pairwise values against the corresponding natural logarithm of geographic distances. Because geographically distant human populations are often also separated by linguistic boundaries, leading to spurious correlations, we performed a partial Mantel test to calculate the partial correlation between linearized *F*_*ST*_/*D*_*est*_ values and human linguistic diversity after controlling for straight-line geographic distance. We caution that simple and partial Mantel tests have been questioned recently for showing inflated type-1 error rate in the presence of spatial autocorrelation, even when a geographic distance matrix is included in the analysis [60]. Thus, the influence of geographic distances and human linguistic diversity on *F*_*ST*_/*D*_*est*_ calculated among *J*. *regia* populations was evaluated with a multiple regression on distance matrices approach [31] using function ‘MRM’ implemented in the “ecodist” R package [61]. The significance of regression coefficients and model *r* ^2^ were estimated using 5,000 permutations.

We investigated a putative correspondence between human linguistic changes and five genetic barriers among common walnut populations detected in Pollegioni et al [3] using the Monmonier’s maximum difference algorithm and Delauney triangulation as implemented in BARRIER software 2.2 [62]. As suggested by Belle and Barbujani [50], each edge of Delauney triangulation was associated with a measure of human linguistic differentiation. We calculated the proportion of Delaunay connections crossed by a statistically significant genetic barrier for each class of linguistic distance. Subsequently, we grouped the indexes of linguistic distance at the level *d*_LAN_ = 4 versus *d*_LAN_ = 0, 1, 2, 3. The proportion of Delaunay connections crossed by a statistically significant genetic barrier was recalculated for the two corresponding groups and the difference in the percentages was tested using the Chi-Square test for a 2x2 contingency table.

## Supporting Information

S1 Fig Geographic location of 39 common walnut populations collected across its Asian range. Kyrgyzstan (1–9), Uzbekistan (10–26), China (27–32), Pakistan (33–34), Tajikistan (35), Iran (36), Turkey (37) and Georgia (38–39). The Silk Road (solid red line) and other trade routes (dotted red line) across the Asian continent were reported as proposed by Francis et al. [25].
(TIF)

S2 Fig UPGMA cluster analysis of 39 common walnut populations based on unbiased Nei’s genetic distance. UPGMA cluster analysis based on unbiased Nei’s [26] genetic distance and 1000 bootstraps for 39 common walnut populations from the species’ Asian range. The number near each node represents the percentage of times when the node occurred among 1000 bootstraps.
(TIF)

S3 Fig Mean genetic distances among walnut populations for each human linguistic distance. Mean genetic distances (D_GEN_) computed as *F*_*ST*_ [27] and *D*_*est*_ [28] values using 14 SSR markers and linguistic distances (D_LAN_) calculated on the basis of Ruhlen’s classification of languages [49] combined with The Ethnologue website [48] among 39 walnut geographic sites. Mean values showing the same letter are not significantly different at *P* ≤ 0.05 according to the post hoc Tukey’s test.
(TIF)

S4 Fig Five statistically significant genetic barriers among 39 common walnut populations identified. Solid red line indicates statistically significant genetic boundaries. The classification of languages into four phyla spoken by human communities in the geographic sampling sites were also reported.
(TIF)

S1 Table Description of 39 common walnut populations sampled in Asia. Number of samples (N), and geographic description for 39 common walnut populations collected across the species’ Asian range [3]. Language name, subgroup, family and phylum spoken by human communities for each geographic sampling site were also reported according to The Ethnologue website [48] and Ruhlen’s classification of languages [49].
(DOCX)

S2 Table Mean percentage of membership (*Qi*) of each common walnut population inferred by STRUCTURE. Mean percentage of membership (*Qi*) of each predefined common walnut population in each of the four (K = 4) clusters and four (K’ = 4) sub-clusters of cluster 2 inferred by STRUCTURE [24]. *Q*-values greater than 0.80 are reported in bold. The number and percentage of walnut genotypes from each population assigned (*Qi* ≥ 0.80) to each of four clusters (K = 4) and four sub-clusters (K’ = 4) were also reported below *Q*-values. Populations and/or individuals with 0.20 < Qi < 0.80 were classified as admixed populations and /or genotypes.
(DOCX)
