# Supplementary figures and images for: Ancient Humans Influenced the Current Spatial Genetic Structure of Common Walnut Populations in Asia
Source: PLoS One. 2015 Sep 2;10(9):e0135980. doi: 10.1371/journal.pone.0135980 (PMC4557929; doi:10.1371/journal.pone.0135980)

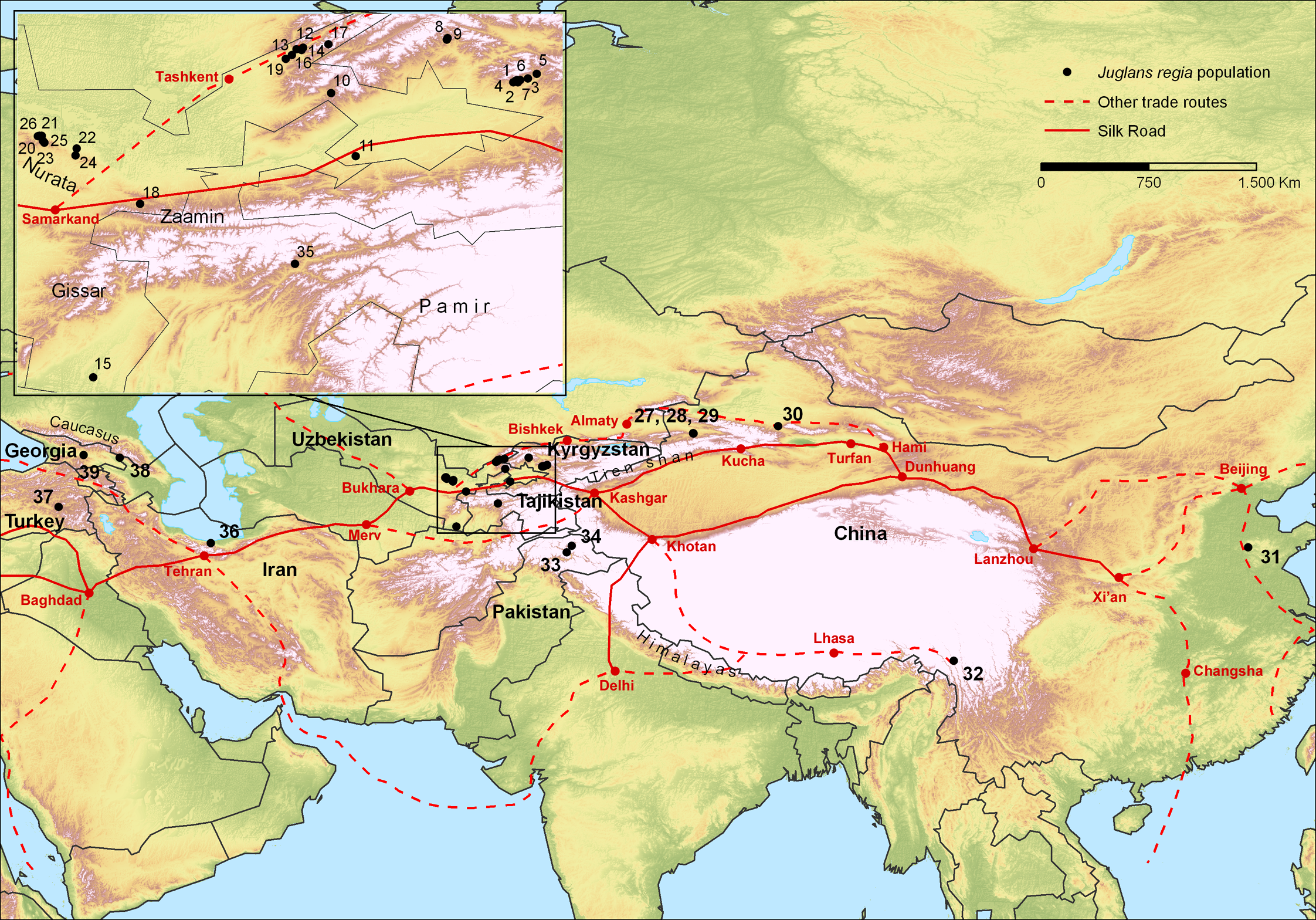

Supplement: S1 Fig — Kyrgyzstan (1–9), Uzbekistan (10–26), China (27–32), Pakistan (33–34), Tajikistan (35), Iran (36), Turkey (37) and Georgia (38–39). The Silk Road (solid red line) and other trade routes (dotted red line) across the Asian continent were reported as proposed by Francis et al. [25]. (TIF) [file pone.0135980.s001.tif]

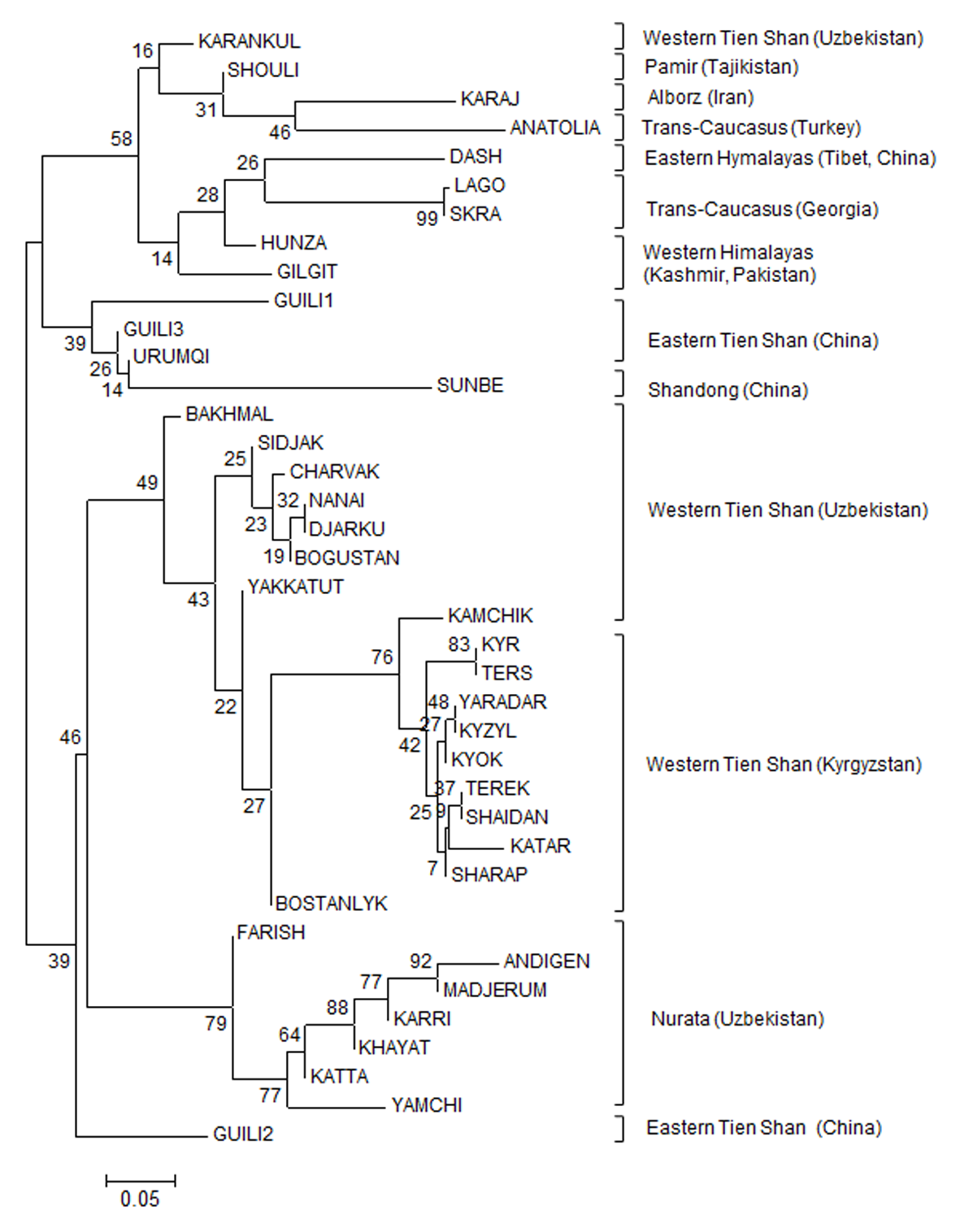

Supplement: S2 Fig — UPGMA cluster analysis based on unbiased Nei’s [26] genetic distance and 1000 bootstraps for 39 common walnut populations from the species’ Asian range. The number near each node represents the percentage of times when the node occurred among 1000 bootstraps. (TIF) [file pone.0135980.s002.tif]

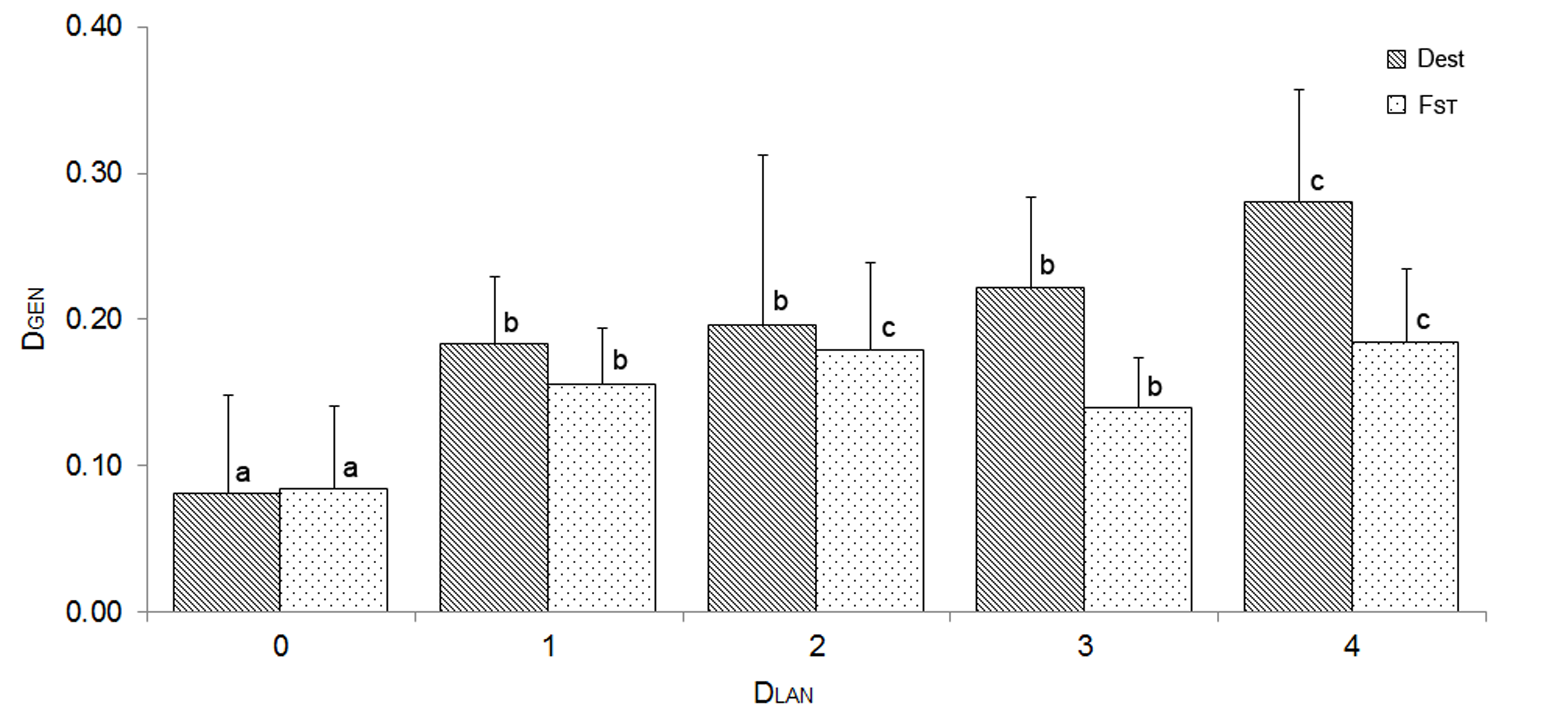

Supplement: S3 Fig — Mean genetic distances (DGEN) computed as F ST [27] and D est [28] values using 14 SSR markers and linguistic distances (DLAN) calculated on the basis of Ruhlen’s classification of languages [49] combined with The Ethnologue website [48] among 39 walnut geographic sites. Mean values showing the same letter are not significantly different at P ≤ 0.05 according to the post hoc Tukey’s test. (TIF) [file pone.0135980.s003.tif]

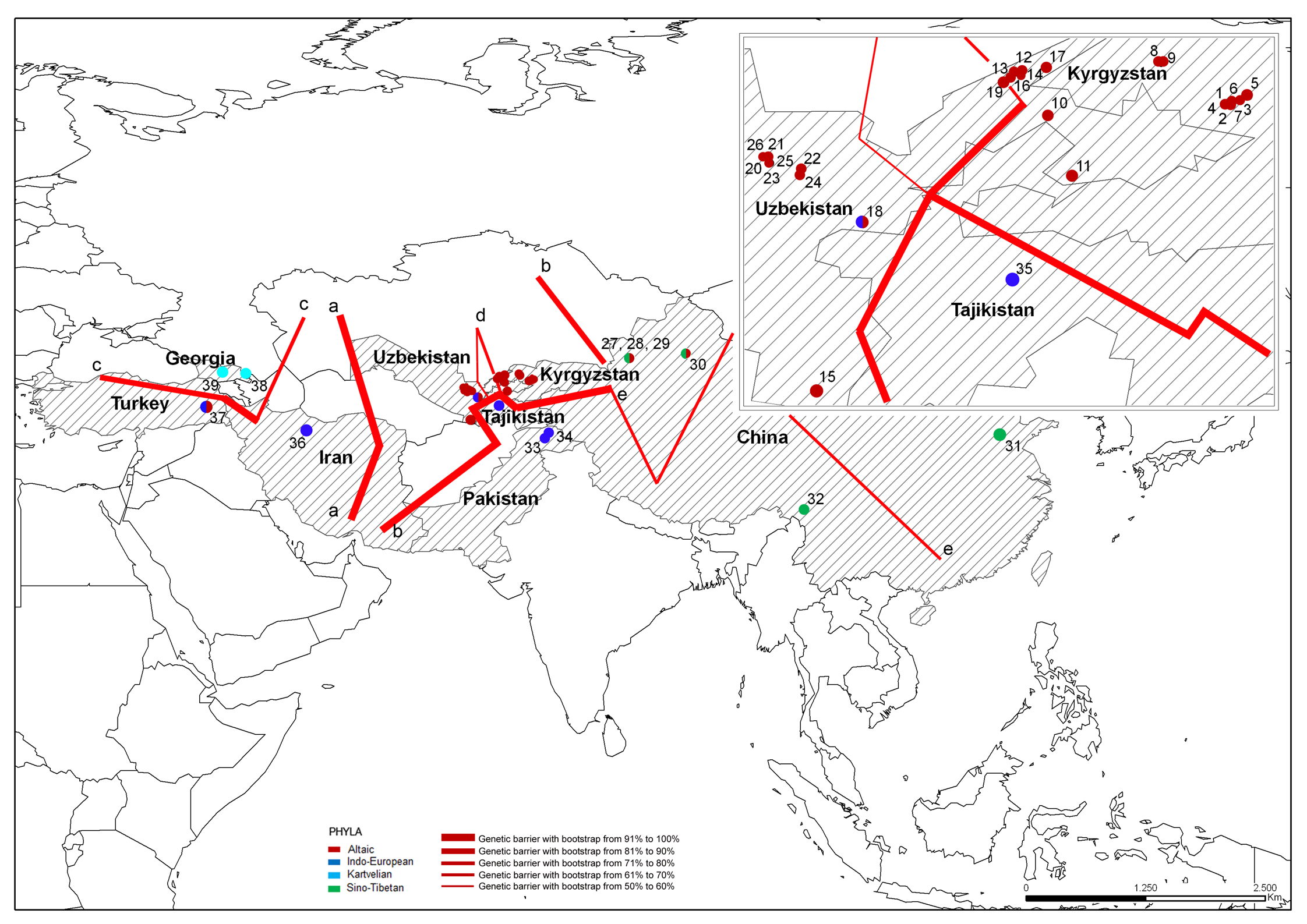

Supplement: S4 Fig — Solid red line indicates statistically significant genetic boundaries. The classification of languages into four phyla spoken by human communities in the geographic sampling sites were also reported. (TIF) [file pone.0135980.s004.tif]
