# Supplementary material for: Ancient Humans Influenced the Current Spatial Genetic Structure of Common Walnut Populations in Asia
Source: PLoS One. 2015 Sep 2;10(9):e0135980. doi: 10.1371/journal.pone.0135980 (PMC4557929; doi:10.1371/journal.pone.0135980)
Supplement: S1 Table — Number of samples (N), and geographic description for 39 common walnut populations collected across the species’ Asian range [3]. Language name, subgroup, family and phylum spoken by human communities for each geographic sampling site were also reported according to The Ethnologue website [48] and Ruhlen’s classification of languages [49]. (DOCX) [file pone.0135980.s005.docx]

**S1 Table.** **Description of 39 common walnut populations sampled in Asia**. Number of samples (N), and geographic description for 39 common walnut populations collected across the species’ Asian range [3]. Language name, subgroup, family and phylum spoken by human communities for each geographic sampling site were also reported according to The Ethnologue website [48] and Ruhlen’s classification of languages [49].

| Country | Province | Mountain | Site  N | Population | Abbreviation | N | Language | Subgroup | Family | Phylum |
| --- | --- | --- | --- | --- | --- | --- | --- | --- | --- | --- |
| Kyrgyzstan | Jalal-Abad | Western Tien Shan | 1 | Ak-Terek | TEREK | 45 | Kyrgyz | Western | Turkic | Altaic |
|  |  | Western Tien Shan | 2 | Sharap | SHARAP | 18 | Kyrgyz | Western | Turkic | Altaic |
|  |  | Western Tien Shan | 3 | Yaradar | YARADAR | 16 | Kyrgyz | Western | Turkic | Altaic |
|  |  | Western Tien Shan | 4 | Shaidan | SHAIDAN | 24 | Kyrgyz | Western | Turkic | Altaic |
|  |  | Western Tien Shan | 5 | Kyzyl-Ungur | KYZYL | 45 | Kyrgyz | Western | Turkic | Altaic |
|  |  | Western Tien Shan | 6 | Katar-Yangak | KATAR | 19 | Kyrgyz | Western | Turkic | Altaic |
|  |  | Western Tien Shan | 7 | Kyok-Sarau | KYOK | 25 | Kyrgyz | Western | Turkic | Altaic |
|  |  | Western Tien Shan | 8 | Kyr-sai ^a^ | KYR | 27 | Kyrgyz | Western | Turkic | Altaic |
|  |  | Western Tien Shan | 9 | Ters-Kolt ^a^ | TERS | 24 | Kyrgyz | Western | Turkic | Altaic |
| Uzbekistan | Namangan | Western Tien Shan | 10 | Kamchik | KAMCHIK | 18 | Northern Uzbek | Eastern | Turkic | Altaic |
|  | Fergana | Western Tien Shan | 11 | Yakkatut | YAKKATUT | 18 | Northern Uzbek | Eastern | Turkic | Altaic |
|  | Tashkent | Western Tien Shan | 12 | Sidjak | SIDJAK | 10 | Northern Uzbek | Eastern | Turkic | Altaic |
|  |  | Western Tien Shan | 13 | Charvak | CHARVAK | 18 | Northern Uzbek | Eastern | Turkic | Altaic |
|  |  | Western Tien Shan | 14 | Nanai | NANAI | 18 | Northern Uzbek | Eastern | Turkic | Altaic |
|  |  | Western Tien Shan | 16 | Bogustan | BOGUSTAN | 20 | Northern Uzbek | Eastern | Turkic | Altaic |
|  |  | Western Tien Shan | 17 | Bostanlyk ^b^ | BOSTANLIK | 18 | Northern Uzbek | Eastern | Turkic | Altaic |
|  |  | Western Tien Shan | 19 | Karankul | KARANKUL | 36 | Northern Uzbek | Eastern | Turkic | Altaic |
|  | Surkhandarya | Zaamin | 15 | Djarkurgan | DJARKU | 16 | Northern Uzbek | Eastern | Turkic | Altaic |
|  | Jizakh | Gissar | 18 | Bakhmal ^c^ | BAKHMAL | 15 | Tajiki | Western | Iranian | Indo-European ^i^ |
|  |  |  |  |  |  |  | Northern Uzbek | Eastern | Turkic | Altaic |
|  |  | Nurata | 20 | Farish | FARISH | 19 | Northern Uzbek | Eastern | Turkic | Altaic |
|  |  | Nurata | 21 | Andigen | ANDIGEN | 12 | Northern Uzbek | Eastern | Turkic | Altaic |
|  |  | Nurata | 22 | Katta-Bogdan | KATTA | 38 | Northern Uzbek | Eastern | Turkic | Altaic |
|  |  | Nurata | 23 | Khayat | KHAYAT | 16 | Northern Uzbek | Eastern | Turkic | Altaic |
|  |  | Nurata | 24 | Yamchi | YAMCHI | 10 | Northern Uzbek | Eastern- | Turkic | Altaic |
|  |  | Nurata | 25 | Karri | KARRI | 20 | Northern Uzbek | Eastern | Turkic | Altaic |
|  |  | Nurata | 26 | Madjerum ^d^ | MADJERUM | 28 | Northern Uzbek | Eastern | Turkic | Altaic |
| China | Xinjiang | Eastern Tien Shan | 27 | Gongliu-1 ^e^ | GUILI-1 | 34 | Uyghur | Eastern | Turkic | Altaic |
|  |  |  |  |  |  |  | Chinese, Mandarin | - | Sinitic | Sino-Tibetan |
|  |  | Eastern Tien Shan | 28 | Gongliu-2 ^e^ | GUILI-2 | 67 | Uyghur | Eastern | Turkic | Altaic |
|  |  |  |  |  |  |  | Chinese, Mandarin | - | Sinitic | Sino-Tibetan |
|  |  | Eastern Tien Shan | 29 | Gongliu-3 ^e^ | GUILI-3 | 27 | Uyghur | Eastern | Turkic | Altaic |
|  |  |  |  |  |  |  | Chinese, Mandarin | - | Sinitic | Sino-Tibetan |
|  |  | Eastern Tien Shan | 30 | Urumqi | URUMQI | 29 | Uyghur | Eastern | Turkic | Altaic |
|  |  |  |  |  |  |  | Chinese, Mandarin | - | Sinitic | Sino-Tibetan |
|  | Shandong | - | 31 | Sunbè | SUNBE | 19 | Chinese, Mandarin | - | Sinitic | Sino-Tibetan |
|  | Tibet | Eastern Himalayas | 32 | Dashuicun | DASH | 48 | Tibetan | Western | Tibeto-Burman | Sino-Tibetan |
|  |  |  |  |  |  |  | Chinese, Mandarin | - | Sinitic | Sino-Tibetan |
| Pakistan | Gilgit-Baltistan | Western Himalayas | 33 | Gilgit Valley | GILGIT | 21 | Urdu | Central | Indo-Aryan | Indo-European ^i^ |
|  |  | Western Himalayas | 34 | Hunza Valley | HUNZA | 25 | Urdu | Central | Indo-Aryan | Indo-European ^i^ |
| Tajikistan | Karotegin | Pamir | 35 | Shouli | SHOULI | 16 | Tajiki | Western | Iranian | Indo-European ^i^ |
| Iran | Alborz | Alborz | 36 | Karaj | KARAJ | 12 | Persian, Iranian | Western | Iranian | Indo-European |
| Turkey |  | Trans-Caucasus | 37 | Anatolia | ANATOLIA | 19 | Turkish | Southern | Turkic | Altaic |
|  |  |  |  |  |  |  | Luvial ^g^ | - | Anatolian | Indo-European ^i^ |
| Georgia | Kakheti | Trans-Caucasus | 38 | Lagodekh ^f^ | LAGO | 15 | Georgian | - | Karto-Zan | Kartvelian |
|  | Shida Kartli | Trans-Caucasus | 39 | Skra | SKRA | 21 | Georgian | - | Karto-Zan | Kartvelian |
| Total |  |  | - | - |  | 926 |  |  |  |  |

^a^ Sary-Chalek Bioshpere Reserve.

^b^ Ugam-Chatkal National Park.

^c^ Zaamin National Park.

^d^ Nurata Nature Reserve.

^e^ Gongliu Wild Walnut Nature Reserve.

^f^ Lagodekhi Nature Reserve.

^g^ Luvial is an ancient language spoken in a vast area stretching from the Sakarya river basin in northwestern Anatolia to the Euphrates valley in present-day Syria during second and first millennia BC.

^h^ Indo-European is replaced by Indo-Hittite Phylum in Ruhlen’s classification.
