# Supplementary material for: Ancient Humans Influenced the Current Spatial Genetic Structure of Common Walnut Populations in Asia
Source: PLoS One. 2015 Sep 2;10(9):e0135980. doi: 10.1371/journal.pone.0135980 (PMC4557929; doi:10.1371/journal.pone.0135980)
Supplement: S2 Table — Mean percentage of membership (Qi) of each predefined common walnut population in each of the four (K = 4) clusters and four (K’ = 4) sub-clusters of cluster 2 inferred by STRUCTURE [24]. Q-values greater than 0.80 are reported in bold. The number and percentage of walnut genotypes from each population assigned (Qi ≥ 0.80) to each of four clusters (K = 4) and four sub-clusters (K’ = 4) were also reported below Q-values. Populations and/or individuals with 0.20 < Qi < 0.80 were classified as admixed populations and /or genotypes. (DOCX) [file pone.0135980.s006.docx]

**S2 Table.** **Mean percentage of membership (*Qi*) of each common walnut population inferred by STRUCTURE**. Mean percentage of membership (*Qi*) of each predefined common walnut population in each of the four (K = 4) clusters and four (K’ = 4) sub-clusters of cluster 2 inferred by STRUCTURE [24]. *Q*-values greater than 0.80 are reported in bold. The number and percentage of walnut genotypes from each population assigned (*Qi* ≥ 0.80) to each of four clusters (K = 4) and four subclusters (K’ = 4) were also reported below *Q*-values. Populations and/or individuals with 0.20 < Qi < 0.80 were classified as admixed populations and /or genotypes.

|  |  |  |  | K = 4 | | | |  |  | K’ = 4 | | | |  |
| --- | --- | --- | --- | --- | --- | --- | --- | --- | --- | --- | --- | --- | --- | --- |
| Country | ID | N |  | Q1 | Q2 | Q3 | Q4 | Admixed  genotypes |  | Q1 | Q2 | Q3 | Q4 | Admixed  genotypes |
| Kyrgyzstan | 1-TEREK | 45 |  | **0.9020**  40 (88.9%) | 0.0670  1 (2.2%) | 0.0170  (-) | 0.0140  (-) | 4 (8.9%) |  |  |  |  |  |  |
|  | 2-SHARAP | 18 |  | **0.8956**  15 (83.3%) | 0.0110  (-) | 0.0293  (-) | 0.0640  (-) | 3 (16.7%) |  |  |  |  |  |  |
|  | 3-YARADAR | 16 |  | **0.9513**  15 (93.7%) | 0.0140  (-) | 0.0177  (-) | 0.0170  (-) | 1 (6.3%) |  |  |  |  |  |  |
|  | 4-SHAIDAN | 24 |  | **0.9344**  23(95.8%) | 0.0470  1 (4.2%) | 0.0060  (-) | 0.0127  (-) | - |  |  |  |  |  |  |
|  | 5-KYZYL | 45 |  | **0.9710**  44 (97.8%) | 0.0070  (-) | 0.0090  (-) | 0.0130  (-) | 1 (2.2%) |  |  |  |  |  |  |
|  | 6-KATAR | 19 |  | **0.9677**  19 (100%) | 0.0093  (-) | 0.0090  (-) | 0.0140  (-) | - |  |  |  |  |  |  |
|  | 7-KYOK | 25 |  | **0.9530**  24 (96%) | 0.0220  (-) | 0.0140  (-) | 0.0110  (-) | 1 (4%) |  |  |  |  |  |  |
|  | 8-KYR | 27 |  | **0.9470**  25 (92.6%) | 0.0083  (-) | 0.0170  (-) | 0.0277  (-) | 2 (7.4%) |  |  |  |  |  |  |
|  | 9-TERS | 24 |  | **0.9470**  24 (100%) | 0.0110  (-) | 0.0137  (-) | 0.0283  (-) | - |  |  |  |  |  |  |
| Uzbekistan | 10-KAMCHIK | 18 |  | 0.7780  9 (50%) | 0.0417  (-) | 0.1180  (-) | 0.0623  1 (5.5%) | 8 (44.5%) |  |  |  |  |  |  |
|  | 11-YAKKATUT | 18 |  | 0.7060  5 (27.7%) | 0.0390  (-) | 0.2253  (-) | 0.0297  (-) | 13 (72.3%) |  |  |  |  |  |  |
|  | 12-SIDJAK | 10 |  | 0.7291  5 (50%) | 0.0270  (-) | 0.0943  (-) | 0.1496  (-) | 5 (50%) |  |  |  |  |  |  |
|  | 13-CHARVAK | 18 |  | 0.6611  5 (27.7%) | 0.0620  (-) | 0.2289  (-) | 0.0480  (-) | 13 (72.3%) |  |  |  |  |  |  |
|  | 14-NANAI | 18 |  | 0.5433  7 (38.9%) | 0.0823  (-) | 0.2483  (-) | 0.1260  (-) | 11 (61.1%) |  |  |  |  |  |  |
|  | 16-BOGUSTAN | 20 |  | 0.5733  8 (40%) | 0.0190  1 (5%) | 0.3170  (-) | 0.0907  (-) | 11 (55%) |  |  |  |  |  |  |
|  | 17-BOSTANLIK | 18 |  | 0.5323  5 (27.8%) | 0.0200  (-) | 0.3250  1 (5.55%) | 0.1227  1 (5.55%) | 11 (61.1%) |  |  |  |  |  |  |
|  | 19-KARANKUL | 36 |  | 0.1170  1 (2.8%) | 0.6830  21 (58.3%) | 0.1237  (-) | 0.0763  (-) | 14 (38.9%) |  | 0.0584  (-) | 0.0415  (-) | **0.8575**  17 (80.9%) | 0.0427  (-) | 4 (19.1%) |
|  | 15-DJARKU | 16 |  | 0.4842  2 (12.5%) | 0.0813  (-) | 0.3049  (-) | 0.1296  (-) | 14 (87.5%) |  |  |  |  |  |  |
|  | 18-BAKHMAL | 15 |  | 0.4401  4 (28.1%) | 0.1507  1 (8.1%) | 0.3525  3 (20%) | 0.0567  (-) | 7 (43.8%) |  |  |  |  |  |  |
|  | 20-FARISH | 19 |  | 0.1873  2 (10.6%) | 0.0100  (-) | 0.6913  10 (52.6%) | 0.1113  (-) | 7 (36.8%) |  |  |  |  |  |  |
|  | 21-ANDIGEN | 12 |  | 0.0050  (-) | 0.0060  (-) | **0.9833**  12 (100%) | 0.0057  (-) | - |  |  |  |  |  |  |
|  | 22-KATTA | 38 |  | 0.0193  (-) | 0.0170  (-) | **0.9363**  37 (97.4%) | 0.0274  (-) | 1 (2.6%) |  |  |  |  |  |  |
|  | 23-KHAYAT | 16 |  | 0.0060  (-) | 0.0090  (-) | **0.9670**  16 (100%) | 0.0180  (-) | - |  |  |  |  |  |  |
|  | 24-YAMCHI | 10 |  | 0.0340  (-) | 0.0107  (-) | **0.9363**  10 (100%) | 0.0190  (-) | - |  |  |  |  |  |  |
|  | 25-KARRI | 20 |  | 0.0070  (-) | 0.0120  (-) | **0.9720**  20 (100%) | 0.0090  (-) | - |  |  |  |  |  |  |
|  | 26-MADJERUM | 28 |  | 0.0120  (-) | 0.0080  (-) | **0.9670**  28 (100%) | 0.0130  (-) | - |  |  |  |  |  |  |
| China | 27-GUILI-1 | 34 |  | 0.0100  (-) | 0.0510  (-) | 0.0180  (-) | **0.9210**  30 (88.2%) | 4 (11.8%) |  |  |  |  |  |  |
|  | 28-GUILI-2 | 67 |  | 0.0230  (-) | 0.1510  10 (14.9%) | 0.0147  (-) | **0.8113**  57 (85.1%) | - |  | **0.8445**  9 (90%) | 0.006  (-) | 0.0423  (-) | 0.1072  1 (10%) | - |
|  | 29-GUILI-3 | 27 |  | 0.0283  (-) | 0.0163  (-) | 0.0127  (-) | **0.9427**  26 (96.3%) | 1 (3.7%) |  |  |  |  |  |  |
|  | 30-URUMQI | 29 |  | 0.0170  (-) | 0.0730  2 (6.9%) | 0.0217  (-) | **0.8883**  26 (89.6%) | 1 (3.5%) |  |  |  |  |  |  |
|  | 31-SUNBE | 19 |  | 0.0120  (-) | 0.0130  (-) | 0.0060  (-) | **0.9690**  19 (100%) | - |  |  |  |  |  |  |
|  | 32-DASH | 48 |  | 0.0063  (-) | **0.9656**  48 (100%) | 0.0140  (-) | 0.0140  (-) | - |  | **0.9465**  48 (100%) | 0.013 | 0.018 | 0.0225 | - |
| Pakistan | 33-GILGIT | 21 |  | 0.0167  (-) | **0.8756**  21 (100%) | 0.0564  (-) | 0.0514  (-) | - |  | 0.0122  (-) | 0.0090  (-) | 0.2441  4 (19.1%) | 0.7347  17 (80.9%) | - |
|  | 34-HUNZA | 25 |  | 0.0350  (-) | **0.9147**  25 (100%) | 0.0197  (-) | 0.0306  (-) | - |  | 0.0260 (1)  (-) | 0.0153  (-) | 0.0120  (-) | **0.9467**  25 (100%) | **-** |
| Tadjikistan | 35-SHOULI | 16 |  | 0.1476  2 (12.5%) | **0.8001**  13 (81.25%) | 0.0127  (-) | 0.0397  (-) | 1 (6%) |  | 0.0318  (-) | 0.0397 | 0.6351  5 (38.5%) | 0.2935  3 (23%) | 5 (38.5%) |
| Iran | 36-KARAJ | 12 |  | 0.0180  (-) | **0.8652**  12 (100%) | 0.0180  (-) | 0.0987  (-) | - |  | 0.0050  (-) | 0.0140  (-) | 0.7506  8 (66.6%) | 0.2304  2 (16.7%) | 2 (16.7%) |
| Turkey | 37-ANATOLIA | 19 |  | 0.0620  1 (5.3%) | **0.9066**  18 (94.7%) | 0.0183  (-) | 0.0130  (-) | - |  | 0.0110  (-) | 0.0218  (-) | **0.9113**  18 (94.7%) | 0.0558  1 (5.3%) | - |
| Georgia | 38-LAGO | 15 |  | 0.0160  (-) | **0.9580**  15 (100%) | 0.0130  (-) | 0.0130  (-) | - |  | 0.0217  (-) | **0.9513**  14 (93.3%) | 0.013  (-) | 0.0140  (-) | 1 (6.7%) |
|  | 39-SKRA | 21 |  | 0.0090  (-) | **0.9407**  20 (95.2%) | 0.0423  (-) | 0.0080  (-) | 1 (4.8%) |  | 0.0172  (-) | **0.9547**  20 (95.2%) | 0.0143  (-) | 0.0138  (-) | 1 (4.8%) |
